# A Review of Penile Cancer

**DOI:** 10.1155/2009/415062

**Published:** 2010-02-16

**Authors:** A. Rando Sous, M. Pérez-Utrilla Pérez, A. Aguilera Bazán, A. Tabernero Gomez, J. Cisneros Ledo, J. De la Peña Barthel

**Affiliations:** Department of Urology, Hospital Universitario La Paz, 28046 Madrid, Spain

## Abstract

Cancer of the penis is a rare tumour in Europe and mainly affects the elderly patient population. The aim of this paper was to analyse and study the characteristics of this tumour, in our patient population. *Materials and Methods.* A retrospective study was conducted on penile tumours diagnosed and treated in the Urology Department of the Hospital Universitario La Paz, Madrid, in the last ten years. *Results.* A total of 34 patients were diagnosed and treated. The mean age at presentation was 71.27 years. The mean time between symptoms and the first consultation was 12.54 months with a median of 6 months. The most common form of presentation was balanoposthitis (32%) and the most common site in our series was the glans. Partial penectomy was performed in 22 cases, total amputation in 8, and local excision in 3. *Discussion.* Carcinoma of the penis is a pathology which mostly affects elderly patients; in our series, the highest incidence was observed in patients in the group aged 75–84 years. The most common histological type was epidermoid carcinoma in its various forms of presentation. We recorded a mortality of 23%. *Conclusion.* Penile carcinoma is a rare pathology which affects elderly persons and is diagnosed late.

## 1. Introduction

Penile cancer is a malignant tumour in which malignant cells develop on the skin and/or the thin tissues of the penis. Around 4000 cases are diagnosed each year, comprising less than 0.5% of all cancers [1]. It is rare in Europe and the United States, but not in developing countries or in their immigrants to Europe and the USA [2]. The annual incidence during the period 1993–1997 was estimated to be less than 1/100 000 men in the European population. As always, some regions of Western Europe have an incidence higher than 1/100 000 (among these Malta, some regions of Spain, Neuchatel in Switzerland, and Hauth-Rhin in France) [3]. While in the United States, it represents 0.3% to 0.5% of malignant tumours in men [4], it is estimated that for 2008, the United States will have 1250 new cases of penile cancer and other genital cancers in men and an estimated incidence of death of 290 cases [5]. The variation in the world geographical incidence is evident and may be due to differences in hygiene, social, and religious practices [6]. Penile cancer has a peak incidence in men aged over 70 years; around 60% of cases present in men over 65 years [7]. 

The aim of our study was to present our series during the last 10 years (1998–2008) and to analyse the results.

## 2. Materials and Methods

A retrospective study was carried out on a 10-year period on a total of 34 patients treated for penile carcinoma in the Hospital Universitario La Paz Urology Department, between May 1998 and May 2008. The factors analysed were age, history of phimosis and/or circumcision, time between appearance of the lesion and the first consultation, form of presentation, presence of adenopathies, site of the lesion, anatomical pathology results, type of surgery, postoperative complications, lymphadenectomy, hospital stay, follow-up, adjuvant therapy, and survival. 

## 3. Results

**Figure 1:**
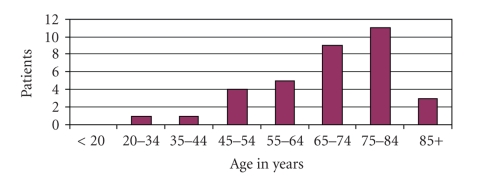
Distribution according to age.

A total of 34 patients were diagnosed and treated in a 10-year period. Our patients were aged between 30 and 93 years, with a mean age of 71.27 years. The distribution according to age is shown in Figure 1.

The mean time between the appearance of symptoms and the first consultation was 12.54 months, with a median of 6 months. In 6 of our patients, the time between appearance and its diagnosis was not shown in the medical record, as they could not remember the date exactly. Ten of the patients had phimosis at the time of diagnosis and seven had been circumcised at some time in their lives. The macroscopic characteristics of the lesions are shown in Table 1. The most common form of presentation was balanoposthitis (32.35%), followed by an ulcerous lesion (17.64%).

The most common site was in the glans. Table 2 shows the surgical technique performed in relation to the tumour stage. Surgery was decided against in one case only as there was an inguinal mass which was considered inoperable. The tumour was an epidermoid carcinoma in 94.17% of the cases, while only one case was verrucous carcinoma and another was reported as diffuse intraepithelial carcinoma.

At the time of diagnosis, 17 patients had clinically palpable inguinal adenopathies and 1 had a pelvic adenopathy diagnosed by CT imaging. After antibiotic treatment, the adenopathies became clinically undetectable in 6 patients; in these cases it was considered that the adenopathies was consequent the concomitant infection. Lymphadenectomy was performed on 10 patients, pelvic and inguinal lymphadenectomy on 6 (hemipelvectomy was carried out in two of these six), and inguinal bilateral only on 4. In the remaining patients, lymph node management was by follow-up. In those cases we made a modified inguinal lymphadenectomy, and the surgical incision was horizontally inguinal.

The mean postoperative hospitalisation time was 2 days. Only 3 patients in our series had adjuvant chemotherapy administered due to evidence of metastatic disease during follow-up and one had radiotherapy, due to the presence of an inoperable inguinal tumour mass. 

Our patients were followedup postoperatively for a mean of 41.77 months and a median 31 (range from 0 to 190 months); postoperative monitoring was not carried out in only 2 patients, as they did not attend the first postsurgical visit. During the follow-up, periodical physical examinations (with biopsy in case of diagnostic uncertainty) and imaging studies (CT, chest X-ray and/or ultrasound) were carried out at three-month intervals for the first two years and then six-month intervals from then on; distant metastases were observed in 5 patients. These patients were referred to the Medical Oncology Department. We recorded a mortality of 23% in our series. All but one of those who died had positive pelvic adenopathies, and 4 of the 8 deceased had received adjuvant treatment, 3 with chemotherapy and one with radiotherapy; the most widely used chemotherapy agents were bleomycin, methotrexate, and cisplatin. The use of these drugs was decision of Oncology Department and we do not find any explication about that why they used one or another medication on the clinical history. The start of adjuvant therapy was 3 weeks after the surgery. 

## 4. Discussion

Penile carcinoma is a neoplasm which mostly affects elderly patients; the usual age for this type of tumour is between the 6th and 7th decade of life [8, 9]. In our series, the mean age was 71.27 years, with a median of 71 years. The highest incidence in the United States between 2001 and 2005 was observed in the group aged 65–74 years, comprising 25.3% of all penile cancers [10]. In our series, the age range with the highest incidence was between 75 and 84 years, with a total of 11 patients (32.35%). The mean time between the appearance of symptoms and the first consultation was 12.54 months, with a median of 6 months, data similar to other series. 

The characteristic form of presentation is an ulcerated lesion, followed by infiltrating/deep lesion and papillary or verrucous lesion [11]. In our series, the most common form of presentation was balanoposthitis with 32.35% while ulcerated lesion occupied fourth place with 11.76%. The most common site in our series was the glans, followed by the prepuce. In two cases, the lesion was even found in both the prepuce and the glans at the time of diagnosis. This is similar to the review by Diz Rodrìguez et al. [12] but different to the localisation reported in other series, where the prepuce was found to be the most common site [13]. 

The most common histopathological type was epidermoid carcinoma in its various forms of presentation [8]. This finding remains similar in our series with 94.17% of cases.

The mean postoperative hospitalisation time was 2 days (range from 0 days to 17 days) and a median of 1 day. This can be explained in our case, as in 33 of the 34 patients, treatment was performed on the primary tumour only during their first hospitalisation and the lymphadenectomy on their second stay. The patient who had the longest hospitalisation time had a simultaneous hemipelvectomy and treatment of the primary tumour, requiring admission to the intensive care unit. In our department, the lymphadenectomy was made in second time, with the intention to reduce the morbility of the first surgery. The mean time between the surgery of the primary tumour and the lymphadenectomy was a month. 

Dissemination of penile cancer presents in stages; infiltration of the inguinal lymph nodes is first diagnosed, followed by the iliac chain and finally distant metastases in less than 10% of cases [14]. In our series, we observed 11.76% of distant metastases during follow-up of our patients. The site of the metastases was lung, bone, liver, and inguinal invasion. We recorded a cancer-specific mortality of 23.53%. The 5-year survival was 82.35%, a survival similar to that reported by the American Cancer Society, who states a relative survival of 78% [15]. At the time of diagnosis of the initial lesion, around 50% of patients have palpable inguinal adenopathies; of these, only half will be tumours as penile cancer is usually infected and causes inflammatory adenopathies [16]. In our series, 50% had adenopathies at the time of diagnosis, data similar to other published series; however, it was due to an inflammatory process in only 35% (6 patients). Among the 10 patients in whom lymphadenectomy was performed, lymph node infiltration was demonstrated in only 4 patients.

## 5. Conclusion

Penile carcinoma is pathology with low incidence in relation to other tumours and is diagnosed late, probably due to ignorance of the disease by our patients. It generally affects elderly men, and so the treatment given would not be aggressive. The overtreatment many times carries on a lot of side effects. Therefore, it would be advisable to run campaigns in the elderly male population recommending early consultation in the case of any change in the penis; however trivial it may seem. 

## Figures and Tables

**Table 1:** Macroscopic characteristics of the lesions.

Form of presentation	Number of patients (%)
Balanoposthitis	11 (32.35)
Verrucous lesion	6 (17.64)
Indurated plaque	4 (11.76)
Ulcerated lesion	4 (11.76)
Phimosis	2 (5.88)
Stony mass	2 (5.88)
Not specified	2 (5.88)
Inguinal adenopathic mass	1 (2.94)
Urethrorrhagia	1 (2.94)
Scrotal abscess	1 (2.94)

Location	

Prepuce	7
Glans	18
Balanopreputial sulcus	6
Body	1
Simultaneous glans-prepuce	2

**Table 2:** Primary treatment according to the Stage of the primary tumour.

Treatment	Tx/T0	Tis	Ta	T1	T2	T3	T unknown
Local excision	—	1		1			1
Penectomy							
Partial	—	1	1	14	6	—	—
Total	—	—	—	2	2	4	—
None/Rejection	—	—	—	—	—	—	—
Decided against	—		—	—	1	—	—
TOTAL	0	2	1	17	9	4	1
